# Ensemble Deep-Learning-Enabled Clinical Decision Support System for Breast Cancer Diagnosis and Classification on Ultrasound Images

**DOI:** 10.3390/biology11030439

**Published:** 2022-03-14

**Authors:** Mahmoud Ragab, Ashwag Albukhari, Jaber Alyami, Romany F. Mansour

**Affiliations:** 1 Information Technology Department, Faculty of Computing and Information Technology, King Abdulaziz University, Jeddah 21589, Saudi Arabia; 2 Centre for Artificial Intelligence in Precision Medicines, King Abdulaziz University, Jeddah 21589, Saudi Arabia; aalbukhari@kau.edu.sa; 3 Mathematics Department, Faculty of Science, Al-Azhar University, Cairo 11884, Egypt; 4 Biochemistry Department, Faculty of Science, King Abdulaziz University, Jeddah 21589, Saudi Arabia; 5 Diagnostic Radiology Department, Faculty of Applied Medical Sciences, King Abdulaziz University, Jeddah 21589, Saudi Arabia; jhalyami@kau.edu.sa; 6 Imaging Unit, King Fahd Medical Research Center, King Abdulaziz University, Jeddah 21589, Saudi Arabia; 7 Department of Mathematics, Faculty of Science, New Valley University, El-Kharga 72511, Egypt; romanyf@sci.nvu.edu.eg

**Keywords:** Clinical Decision Support System, disease diagnosis, medical imaging, Deep Learning, Machine Learning, image processing

## Abstract

**Simple Summary:**

In the literature, there exist plenty of research works focused on the detection and classification of breast cancer. However, only a few works have focused on the classification of breast cancer using ultrasound scan images. Although deep transfer learning models are useful in breast cancer classification, owing to their outstanding performance in a number of applications, image pre-processing and segmentation techniques are essential. In this context, the current study developed a new Ensemble Deep-Learning-Enabled Clinical Decision Support System for the diagnosis and classification of breast cancer using ultrasound images. In the study, an optimal multi-level thresholding-based image segmentation technique was designed to identify the tumor-affected regions. The study also developed an ensemble of three deep learning models for feature extraction and an optimal machine learning classifier for breast cancer detection. The study offers a means of assisting radiologists and healthcare professionals in the breast cancer classification process.

**Abstract:**

Clinical Decision Support Systems (CDSS) provide an efficient way to diagnose the presence of diseases such as breast cancer using ultrasound images (USIs). Globally, breast cancer is one of the major causes of increased mortality rates among women. Computer-Aided Diagnosis (CAD) models are widely employed in the detection and classification of tumors in USIs. The CAD systems are designed in such a way that they provide recommendations to help radiologists in diagnosing breast tumors and, furthermore, in disease prognosis. The accuracy of the classification process is decided by the quality of images and the radiologist’s experience. The design of Deep Learning (DL) models is found to be effective in the classification of breast cancer. In the current study, an Ensemble Deep-Learning-Enabled Clinical Decision Support System for Breast Cancer Diagnosis and Classification (EDLCDS-BCDC) technique was developed using USIs. The proposed EDLCDS-BCDC technique was intended to identify the existence of breast cancer using USIs. In this technique, USIs initially undergo pre-processing through two stages, namely wiener filtering and contrast enhancement. Furthermore, Chaotic Krill Herd Algorithm (CKHA) is applied with Kapur’s entropy (KE) for the image segmentation process. In addition, an ensemble of three deep learning models, VGG-16, VGG-19, and SqueezeNet, is used for feature extraction. Finally, Cat Swarm Optimization (CSO) with the Multilayer Perceptron (MLP) model is utilized to classify the images based on whether breast cancer exists or not. A wide range of simulations were carried out on benchmark databases and the extensive results highlight the better outcomes of the proposed EDLCDS-BCDC technique over recent methods.

## 1. Introduction

Breast cancer is one of the most common cancers reported amongst women and is a primary contributor to cancer-related deaths around the world. Early diagnoses of breast cancer can enhance the patient’s quality of life and also increase their survival rate. Further, the mortality rate of the affected patients can also be reduced [1]. The ultrasonography technique is commonly employed in the diagnosis of breast cancer due to its convenience, painless operation and efficient real-time performance [2]. However, the ultrasonic instruments possess high sensitivity, which makes the tissues of the environment in the human body vulnerable. This also results in a massive amount of speckle noise that interferes with doctors’ diagnoses [3]. At present, ultrasound methods are preferred in the diagnosis of breast cancer based on medical expertise. To be specific, ultrasound is involved in the classifications and marks of breast lesions. The ultrasound procedure can be prescribed in this following scenario: the doctor uses an ultrasound instrument to find a better angle and demonstrates the lesion clearly on the screen. Then, they keep the probe fixed for a long period of time using one hand while another hand is used to measure and mark the lesion on the screen [4,5]. In the abovementioned procedure, automatic tracking of the region of interest (lesions) and classification (malignant or benign) are in huge demand for breast lesion detection in USIs.

Computer-Aided Diagnosis (CAD) systems are widely employed in the classification and detection of tumors in breast USIs. This type of system is strongly recommended among radiotherapists for recognizing disease prognoses and breast tumors. As per the literature, the statistical method [6] has been mainly utilized in the analysis of the extracted features such as posterior acoustic attenuation, lesion shape, margin, and homogeneity. However, the recognition of the margins and shapes of lesions is complex in USIs [7]. In addition, Machine Learning (ML) methods have been widely used in both the analysis and classification of lesion-based handcrafted textures and morphological features of tumors [8]. The extraction of features is, however, still largely based on medical expertise. The struggles of researchers for hand-crafted features resulted in the development of new algorithms, such as the Deep Learning (DL) algorithm, that can learn the features automatically from information, especially information that is effective in terms of extracting nonlinear features from the data. The DL model is a promising candidate in the classification of USIs, where the recognition of patterns cannot be hand-engineered with ease [9]. Several research studies, using the DL approach, leverage the idea of a pretrained Convolution Neural Network (CNN) to categorize the tumors in breast USIs [10].

In the current study, an Ensemble Deep-Learning-Enabled Clinical Decision Support System for Breast Cancer Diagnosis and Classification (EDLCDS-BCDC) technique was developed using USIs. The proposed EDLCDS-BCDC technique involves a Chaotic Krill Herd Algorithm (CKHA) with Kapur’s Entropy (KE) technique used for the image segmentation process. Moreover, an ensemble of three deep learning models, namely VGG-16, VGG-19, and SqueezeNet, is used for feature extraction. Furthermore, Cat Swarm Optimization (CSO) with the Multilayer Perceptron (MLP) model is also utilized to classify the images in terms of whether breast cancer exists or not. Extensive experimental analysis was conducted on benchmark database and the results of the EDLCDS-BCDC technique were examined under distinct measures.

## 2. Related Works

Badawy et al. [11] proposed a system based on combined Deep Learning (DL) and Fuzzy Logic (FL) for the automated Semantic Segmentation (SS) of tumors in Breast Ultrasound (BUS) images. The presented system comprises two stages, namely CNN-based SS and FL-based preprocessing. A total of eight common CNN-based SS methods was employed in this work. Almajalid et al. [12] designed a segmentation architecture-based DL framework called U-net for BUS images. U-net is a type of CNN framework that was developed for the segmentation of life science images containing constrained trained data. Yousef Kalaf et al. [13] presented an architecture for the classification of breast cancer with an attention mechanism in an adapted VGG16 framework. The adapted attention model distinguishes between features of the background and targeted lesions in ultrasound image. In addition, an ensemble of loss function was presented; this involved an integration of the logarithm of hyperbolic cosine loss and binary cross-entropy in order to enhance the methodological discrepancy between labels and lesion classification.

Cao et al. [14] conducted a systematic evaluation of the efficiency of a number of current advanced object classification and detection approaches for breast lesion CAD. Then, they estimated distinct DL frameworks and implemented a complete research work on the recently gathered data set. Tanaka et al. [15] designed a CAD scheme to classify benign and malignant tumors using ultrasonography-based CNN. Next, an ensemble network was created in this study by integrating two CNN architectures (VGG192 and ResNe1523). Afterwards, the balanced trained data were fine-tuned using data extension, a common method to synthetically generate new samples from the original. These data were further utilized in a mass level classification technique that enables CNN in the classification of mass with each view. 

Qi et al. [16] developed an automatic breast cancer diagnostics system to increase the accuracy of diagnosis. The scheme, which can be installed on smartphones, takes a picture of the ultrasound report as input, and performs diagnoses on all the images. The presented method comprises three subsystems. Initially, the noise in the captured images is reduced and high-quality images are reconstructed. Next, the initial subsystem is designed according to a stacked Denoising Autoencoder (DAE) framework and Generative Adversarial Network (GAN). Next, the image is classified in terms of whether it is malignant or non-malignant; DCCN is applied to extract the high-level features from the image. At last, anomalies in the system performance are detected, which further reduces the False-Negative Rate (FNR). 

## 3. The Proposed Model

**Figure 1:**
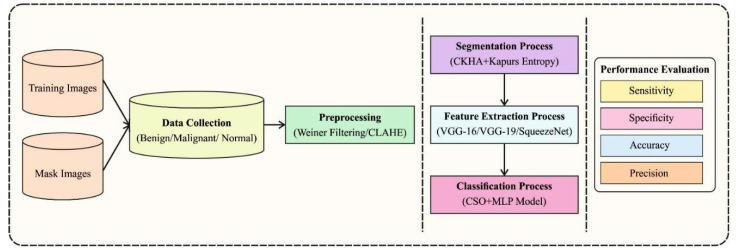
Overall process of the EDLCDS-BCDC technique.

The current study developed a novel EDLCDS-BCDC technique to identify the existence of breast cancer using USIs. In this technique, the pre-processing of USIs primarily occurs in two stages, namely noise elimination and contrast enhancement. Subsequently, CKHA-KE-based image segmentation with ensemble DL-based feature extraction processes are performed. Finally, CSO-MLP model is utilized to classify the images in terms of whether breast cancer exists or not. Figure 1 illustrates the overall process of the EDLCDS-BCDC technique.

### 3.1. Pre-Processing

In this primary stage, the USIs are pre-processed, which involves the noise being removed using the WF technique. Noise extraction is an image pre-processing approach in which the features of an image, corrupted by noise, are enhanced. The adaptive filter is a particular case in which the denoising process is fully dependent upon the noise content that is locally present in the image. Assume that the corrupted images are defined as $\hat{I}\left(x,y\right)$, the noise variance through which the whole point is demonstrated is $\sigma_{y}^{2},$ the local mean is provided as $\hat{\mu_{L}}$ about a pixel window, and local variance from the window is represented as ${\hat{\sigma }}_{y}^{2}$. Then, the probable technique of denoising an image can be demonstrated as follows [17]:

(1) $$\hat{\hat{I}}=\hat{I}\left(x,y\right)-\frac{\sigma_{y}^{2}}{{\hat{\sigma }}_{y}^{2}}\left(\hat{I}\left(x,y\right)-\hat{\mu_{L}}\right)$$

At this point, the noise variance across the image becomes equivalent to zero, $\sigma_{y}^{2}=0=>\hat{\hat{I}}=\hat{I}\left(x,y\right)$. Once the global noise variance becomes lesser while the local variance becomes greater than global variance, the ration is almost equivalent to one.

If ${\hat{\sigma }}_{y}^{2}\gg \sigma_{y}^{2}$, then $\hat{\hat{I}}=\hat{I}\left(x,y\right)$. The high local variance illustrates the occurrence of an edge from the assumed image window. In this case, once the local and global variances match with each other, then the formula is revamped as follows: $\hat{\hat{I}}=\hat{\mu_{L}}\;as\;{\hat{\sigma }}_{y}^{2}\approx \sigma_{y}^{2}$.

It can be an average intensity from a usual region. Furthermore, the contrast is improved with the help of the CLAHE technique [18]. It is an extended version of an adaptive histogram equalization in which the contrast amplification is limited, so as to minimize the noise amplification issue. In CLAHE, the contrast in the neighborhood of a provided pixel value increases, which is offered by the slope of transformation function. It functions on small regions in the image, which are named as ‘tiles’, instead of the whole image. The adjacent tiles are integrated using bilinear interpolation to eliminate the artificial boundary. It can be employed to increase the contrast level of the image.

### 3.2. CKHA-KE Based Image Segmentation

Next, the infected lesion areas are segmented with the help of CKHA-KE technique. The KE technique is applied to determine the optimal threshold value, t. In general, t takes values between 1 and 255 (for 8-bit depth images) and splits an image into $E_{0}$ and $E_{1}$ to maximize the succeeding function [19]:

(2) $$F\left(t\right)=E_{0}+E_{1}\;\;\;\;$$

(3) $$E_{0}=-\overset{t-1}{\underset{i=0}{\sum }}\frac{X_{i}}{T_{0}}\times \ln\;\frac{X_{i}}{T_{0}},X_{i}=\frac{N_{i}}{T},T_{0}=\overset{t-1}{\underset{i=0}{\sum }}X_{i}\;\;\;\;$$

(4) $$E_{1}=-\overset{L-1}{\underset{i=t}{\sum }}\frac{X_{i}}{T_{1}}\times \ln\;\frac{X_{i}}{T_{1}},X_{i}=\frac{N_{i}}{T},T_{1}=\overset{t-1}{\underset{i=1}{\sum }}X_{i},\;\;\;\;$$

$N_{i}$ represents the number of pixels with gray values, represented by i, and T denotes the number of pixels in an image. Equation (1) is adapted easily to find a multiple-threshold value that separates the image into homogenous regions, where it can be redeveloped. Consider a gray image with an intensity value within [0, L−1], then the algorithmic search for finding the n optimum threshold value $\left[t_{0},\;t_{1},\;t_{2},\;\ldots \ldots \;t_{n}\right]$ that subdivides the image to $\left[E_{0},\;E_{1},\;E_{2},\;\ldots \ldots ,\;E_{n}\right]$ to maximize the subsequent function is as follows:

(5) $$F\left(t_{0},\;t_{1},\;t_{2},\;\ldots \ldots \;t_{n}\right)=E_{0}+E_{1}+E_{2}+\cdots \cdots \cdots +E_{n}\;\;$$

(6) $$E_{0}=-\overset{t_{0}-1}{\underset{i=0}{\sum }}\frac{X_{i}}{T_{0}}\times \ln\;\frac{X_{i}}{T_{0}},X_{i}=\frac{N_{i}}{T},T_{0}=\overset{t_{1}-1}{\underset{i=0}{\sum }}X_{i}\;\;$$

(7) $$E_{1}=-\overset{t_{1}-1}{\underset{i=t_{0}}{\sum }}\frac{X_{i}}{T_{1}}\times \ln\;\frac{X_{i}}{T_{1}},X_{i}=\frac{N_{i}}{T},T_{1}=\overset{t_{1}-1}{\underset{i=t_{0}}{\sum }}X_{i}\;$$

(8) $$E_{2}=-\overset{t_{2}-1}{\underset{i=t_{1}}{\sum }}\frac{X_{i}}{T_{2}}\times \ln\;\frac{X_{i}}{T_{2}},X_{i}=\frac{N_{i}}{T},T_{2}=\overset{t_{2}-1}{\underset{i=t_{1}}{\sum }}X_{i}\;\;\;$$

(9) $$E_{n}=-\overset{L-1}{\underset{i=t_{n}}{\sum }}\frac{X_{i}}{T_{n}}\times \;\ln\;\frac{X_{i}}{T_{n}},X_{i}=\frac{N_{i}}{T},T_{n}=\overset{L-1}{\underset{i=t_{n}}{\sum }}X_{i}$$

In order to detect an optimal threshold value for KE, CKHA is derived.

Having idealized on the swarm performance of krill, KHA [20], a meta-heuristic optimization method, is used in resolving optimization problems. In KH, the place is mostly affected by three activities, namely:

i. Drive affected by another krill;
ii. Foraging act;
iii. Physical diffusion.

In KHA, the Lagrangian method is utilized in the existing search space in Equation (10):

(10) $$\frac{dX_{i}}{dr}=N_{i}+F_{i}+D_{i}\;\;\;\;$$

where $N_{i}$ implies the motion created by other krill individuals; $F_{i}$ signifies the foraging motion; and $D_{i}$ is an arbitrary diffusion of the $i^{th}$ krill individual.

A primary one, and its direction, $\alpha_{i}$, is obviously known by the subsequent parts, such as target, local, and repulsive effects. Their brief explanation is given herewith:

(11) $$N_{i}^{new}=N^{\max\;}\alpha_{i}+\omega_{n}N_{i}^{old}\;\;$$

$N^{\max\;},$ $\omega_{n}$ and $N_{i}^{old}$ demonstrate the maximal speed, inertia weight, and final motion, respectively.

The secondary one is computed by two modules, namely the food place and its preceding experience. In order to achieve the $i^{th}$ krill, it could be idealized as follows:

(12) $$F_{i}=V_{f}\beta_{i}+\omega_{f}F_{i}^{old}\;$$

where

(13) $$b_{i}=b_{i}^{food}+b_{i}^{best}$$

and $V_{f}$ refers to the foraging speed, $\omega_{f}$ defines the inertia weight, and $F_{i}^{old}$ represents the final one.

The tertiary part is an essential aspect in arbitrary procedures. It can be calculated based on the maximal diffusion speed and an arbitrary directional vector. Its formulation is given herewith:

(14) $$D_{i}=D^{\max\;}\delta \;$$

where $D^{\max}$ denotes the maximal diffusion speed whereas δ indicates the arbitrary directional vector and their arrays are arbitrary numbers. At this point, the place from KH in r to r+Δr can be expressed as follows:

(15) $$X_{i}\left(t+Dt\right)=X_{i}\left(t\right)+Dt\frac{dX_{i}}{dr}$$

The CKHA technique is derived by incorporating the chaotic concepts into KHA. In this work, a 1-D chaotic map was incorporated in the CKHA design.

### 3.3. Ensemble Feature Extraction

During the feature extraction process, an ensemble of DL models are used, encompassing three approaches, namely VGG-16, VGG-19, and SqueezeNet. The three vectors can be derived as given herewith:

(16) $$f_{VGG16\times m}=\left\{VGG16_{1\times 1},\;VGG16_{1\times 2},\;VGG16_{1\times 3},\;\cdots ,\;VGG16_{1\times n}\right\}\;\;\;\;\;\;\;\;\;\;\;$$

(17) $$f_{VGG19\times m}=\left\{VGG19_{1\times 1},\;VGG19_{1\times 2},\;VGG19_{1\times 3},\;\cdots ,\;VGG19_{1\times n}\right\}\;\;\;\;\;$$

(18) $$f_{\mathrm{SQN}1\times p}=\left\{SQN_{1\times 1},\;SQN_{1\times 2},\;SQN_{1\times 3},\;\cdots ,\;SQN_{1\times n}\right\}\;\;$$

Furthermore, the extracted feature is merged in a single vector:

(19) $$Fused\left(features\;vector\right)1\times q=\overset{3}{\underset{i=1}{\sum }}\{fVGG16_{1\times n},fVGG19_{1\times m},fSQN_{1\times p}\}$$

whereas f represents the fused vector (1×1186). The entropy is employed on the feature vector for selecting the optimum feature according to the score. The FS method is explained arithmetically in Equations (16)–(19). Entropy $B_{He}$ is utilized in the selection of 1186 score-based features from 7835 features as defined below:

(20) $$B_{He}=-NHe_{b}\overset{n}{\underset{i=1}{\sum }}p\left(f_{i}\right)$$

(21) $$F_{select}=B_{He}\left(\max\left(f_{i},\;1186\right)\right)\;$$

In Equations (20) and (21), $F_{select}$ represents the number of features chosen, N denotes the total number of features, and p characterizes the feature probability. The last chosen feature is given to the classifier to differentiate the normal and breast cancer images.

#### 3.3.1. VGG-16 and VGG-19

Simonyan and Zisserman 2014 presented VGG, a sort of CNN framework. The VGG framework won the ILSVR (ImageNet) competition in 2014. The framework enhances the AlexNet framework by replacing kernel-sized filter in which 11 represents the initial convolution layer whereas 5 denotes the next convolutional layer, with numerous small 2 × 2 filters in the max-pooling layer and 3 × 3 kernel-sized filters at the convolution layer consecutively. Finally, it has two FC layers and an activation function softmax/sigmoid for the output. The familiar VGG models are VGG16 and VGG19. Between these, the VGG19 model comprises 19 layers whereas the VGG-16 model comprises 16 layers. The major distinction between the models is the existence of an additional layer at three convolution blocks of the VGG19 model.

#### 3.3.2. SqueezeNet

**Figure 2:**
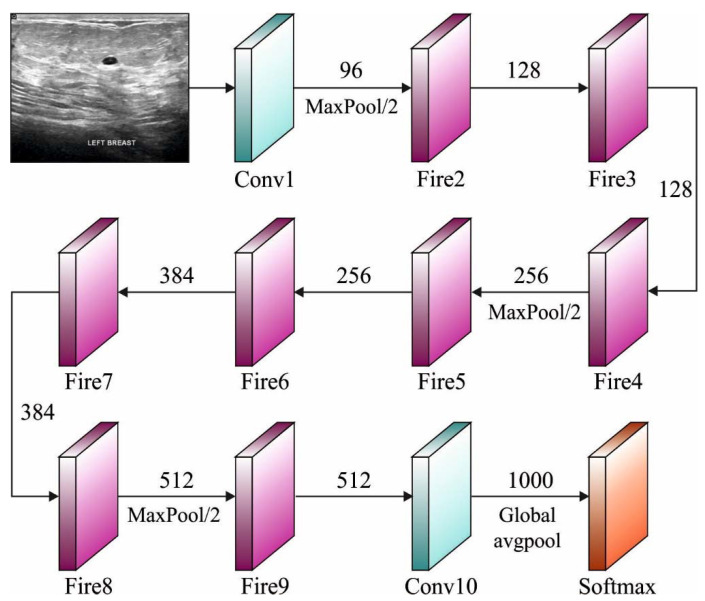
SqueezeNet architecture.

Squeezenet is a kind of DNN that comprises 18 layers and is mainly utilized in image processing and computer vision programs. The primary goals and the objectives of the researchers, in the development of SqueezeNet, are to construct a small NN that comprises fewer parameters and to allow easy transfer through a computer network (requiring less bandwidth). Further, it should also fit into computer memory easily (requiring less memory). The first edition of this framework was executed on top of a DL architecture called Caffe [21]. After a short period of time, the authors started utilizing this framework in many publicly available DL architectures. Firstly, SqueezeNet was labelled, in which it was compared against AlexNet. Both AlexNet and SqueezeNet are two distinct DNN frameworks yet have one common feature, namely accuracy, when estimating the ImageNet image data set. Figure 2 demonstrates the structure of SqueezeNet.

The primary objective of SqueezeNet is to achieve high accuracy using less parameters. To accomplish this objective, three processes are used. Primarily, a 3 × 3 filter is substituted by a 1 × 1 filter with less parameters. Next, the input channel count can be minimized to 3 × 3 filters. At last, the subsampled operation is carried out at the final stages to create a convolutional layer with a large activation function. SqueezeNet is mainly based on the concept of an Inception module [22] to design a Fire module with a squeeze layer and an expansion layer. The fire module comprises a squeeze convolution layer (which has only 1 × 1 filters) that feeds into an expansion layer with a mix of 1 × 1 and 3 × 3 convolutional filters.

### 3.4. Optimal MLP Classifier

Finally, the generated feature vectors are passed onto MLP classifier to allot proper class labels. Perceptron is a simple ANN framework that depends on a slight distinct artificial neuron called the Linear Threshold Unit (LTU) or the Threshold Logic Unit (TLU). The input and output of the cells are numbers whereas all the values are related to weight. TLU evaluates the weighted sum of the input as given below:

(22) $$z=w_{1}x_{1}+w_{2}x_{2}+\ldots +w_{n}x_{n}=x^{\tau_{W}}\;\;\;$$

Later, a step function is employed for that sum and the outcome is viewed as the output:

(23) $$h_{w}\left(x\right)=step\left(z\right)$$

However, $z=x^{\tau_{W}}.$ The perceptron is simply made up of a single layer of TLUs that are interconnected to each input. Once the neuron in a layer is interconnected, it is named as a dense layer or a fully connected layer. The perceptron is stacked by several perceptrons. The resultant ANN is otherwise called the MLP. It is composed of a TLU or a hidden layer in which the ones that pass through are input layers, and other last are output layers. In order to train the MLPS, the BP training approach is utilized to compute the gradient automatically. To optimally adjust the weight values of the MLP model, the CSO algorithm is applied. The CSO algorithm is stimulated from two characteristics of cats, namely the Seeking Model (SM) and Tracking Mode (TM). In the CSO algorithm, the cats possess the locations comprising the D-dimension, the velocity of the dimensions, the fitness value that denotes the inclusion of the cat into the fitness function, and the flag to detect the occurrence of SM or TM. The end solution is determined through the optimal location of the cat and it sustains the optimal ones until the algorithm is terminated [23].

To model the characteristics of cats in the durations of their resting and alert states, SM is used. It includes four major variables such as SMP, SRD, CDC, and SPC. The procedure involved in SM is listed herewith:

Step l: Create j copies of the current location of $cat_{k},$ where j= SMP. When the SPC value is calculated to be true, assume j= (SMP − 1). Then, retain the current location of the candidate.

Step 2: For all copies based on CDC, arbitrarily subtract the current values of the SRD percent and substitute it with previous values.

Step 3: Determine the Fitness Value (FS) for every candidate point.

Step 4: When every FS is non-identical, determine the selection possibility of all the candidate points or else consider the selection possibility of candidate points as ‘1’.

Step 5: Determine the fitness function for every cat. When the fitness function for every cat is identical, then the probability of choosing a cat becomes 1; otherwise, the probability $P_{i}$ can be determined as follows.

(24) $$P_{i}=\frac{\left|F_{i}-F_{b}\right|}{F_{\max\;}-F_{\min\;}}\;\;\;\;$$

where *Fi* indicates the fitness value of a cat, $F_{\max}$ represents the maximum fitness value of cats, $F_{\min}$ denotes the minimal fitness value of the cat, $F_{b}=F_{\max}$ for minimization problems, and $F_{b}=F_{\min}$ for maximization problems.

TM is the next mode of CSO algorithm where the cats aim at tracking their food as well as their targets. The process is listed as follows:

Step 1: Upgrade the velocity of all the dimensions based on Equation (25).

Step 2: Ensure whether the velocity falls inside the range of higher velocity. When the new velocity is above the range, it is considered as equivalent to the limit:

(25) $$V_{k,d}=V_{k,d}+r_{1}c_{1}\left(X_{best,d}-X_{k,d}\right)\;\;\;\;\;\;\;\;$$

Step 3: Upgrade the position of $cat_{k}$ according to (26):

(26) $$x_{k,d}=X_{k,d}+V_{k,d}\;$$

$X_{bestd}$ denotes the location of the cat with optimal fitness and $X_{k,d}$ implies the location of $cat_{k}$; $c_{1}$ denotes the acceleration coefficient to extend the velocity of the cat when moving into the solution space.

## 4. Performance Validation

**Figure 3:**
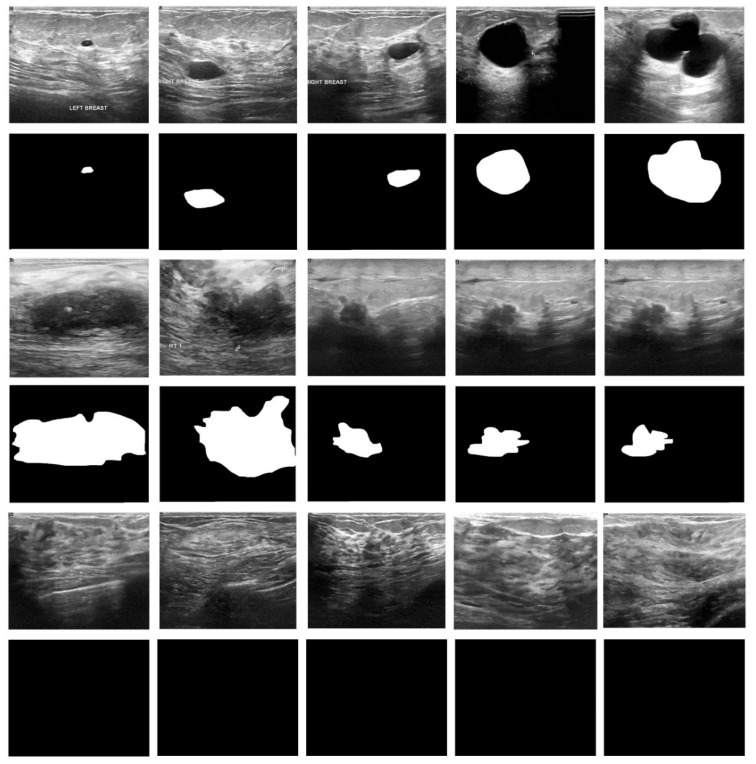
Sample and ground truth images (benign/malignant/normal).

**Figure 4:**
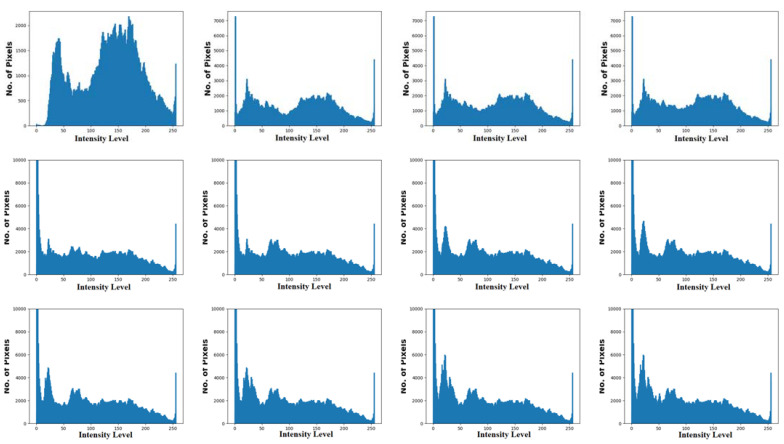
Histogram of the images.

The proposed model was implemented on a PC with the following configuration: Intel i5, 8th generation PC with 16GB RAM, MSI L370 Apro, and Nividia 1050 Ti4 GB. The researchers used Python 3.6.5 along with pandas, sklearn, Keras, Matplotlib, TensorFlow, opencv, Pillow, seaborn and pycm. The experimental analysis was conducted for the EDLCDS-BCDC technique using the benchmark Breast Ultrasound Dataset [24], which comprises 133 images classified as normal, 437 images classified as benign, and 210 images classified as malignant. The dataset holds 780 images sized in the range of 500×500 pixels. Figure 3 shows the input images along with ground truth images. The first, third, and fifth rows represent the original mammogram images. Next, the respective ground truth images are given in the consecutive second, fourth, and sixth images. Furthermore, Figure 4 includes a histogram of the images (for the input images given in the first, third, and fifth rows in Figure 3).

**Figure 5:**
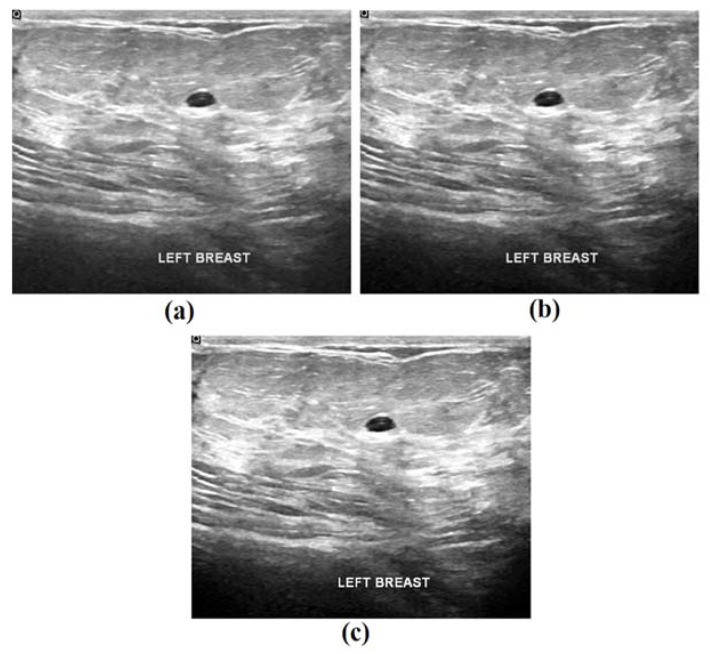
Sample visualization results: (**a**) original image; (**b**) noise-removed image, and (**c**) contrast-enhanced image.

Figure 5 illustrates the sample visualization results of the proposed model during the preprocessing stage. For a given input image, the corresponding noise was removed and the contrast-enhanced images are depicted in the figure. It is evident that the quality of these images was considerably improved in this preprocessing stage.

Table 1 exhibits the overall breast cancer classification analysis results accomplished using the EDLCDS-BCDC technique under several epochs and different measures such as $sens_{y}$, $spec_{y}$, $prec_{n},$ and $accu_{y}$. The table values imply that the proposed EDLCDS-BCDC technique accomplished the maximum breast cancer classification results in all the aspects considered for the study.

Table 2 show the overall breast cancer classification outcomes achieved by the proposed EDLCDS-BCDC technique under several epochs. The results represent the enhanced classifier results for the EDLCDS-BCDC technique under every epoch. For instance, with 250 epochs, the EDLCDS-BCDC technique attained $sens_{y}$, $spec_{y}$, $prec_{n},$ and $accu_{y}$ values of 96.01%, 97.95%, 95.39%, and 97.52%, respectively. Similarly, with 750 epochs, the presented EDLCDS-BCDC technique obtained $sens_{y}$, $spec_{y}$, $prec_{n},$ and $accu_{y}$ values of 95.35%, 97.38%, 93.93%, and 96.75%, respectively. Moreover, with 1500 epochs, the proposed EDLCDS-BCDC technique attained $sens_{y}$, $spec_{y}$, $prec_{n},$ and $accu_{y}$ values of 97.15%, 97.35%, 94.74%, and 96.92%, respectively.

**Figure 6:**
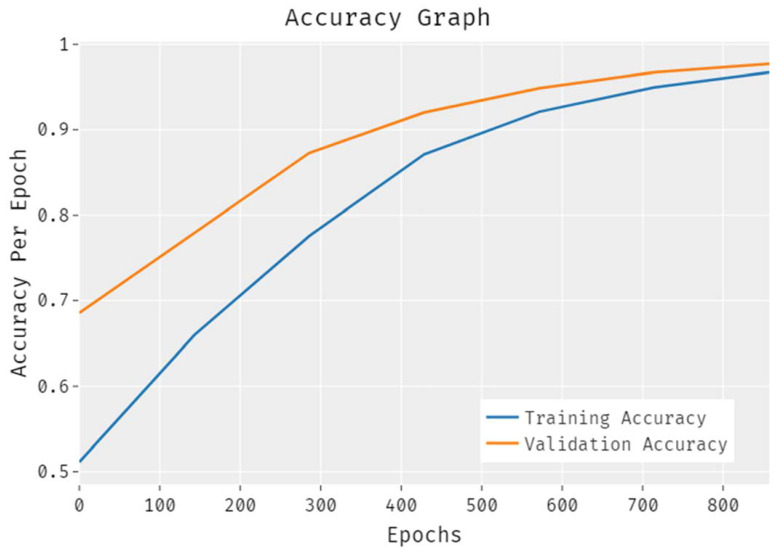
Accuracy analysis results for the EDLCDS-BCDC technique.

The results from the accuracy analysis of the EDLCDS-BCDC technique conducted on the test data are illustrated in Figure 6. The results demonstrate that the proposed EDLCDS-BCDC system accomplished an improved validation accuracy as compared to the training accuracy. Further, the accuracy values were also found to be saturated with the number of epochs.

**Figure 7:**
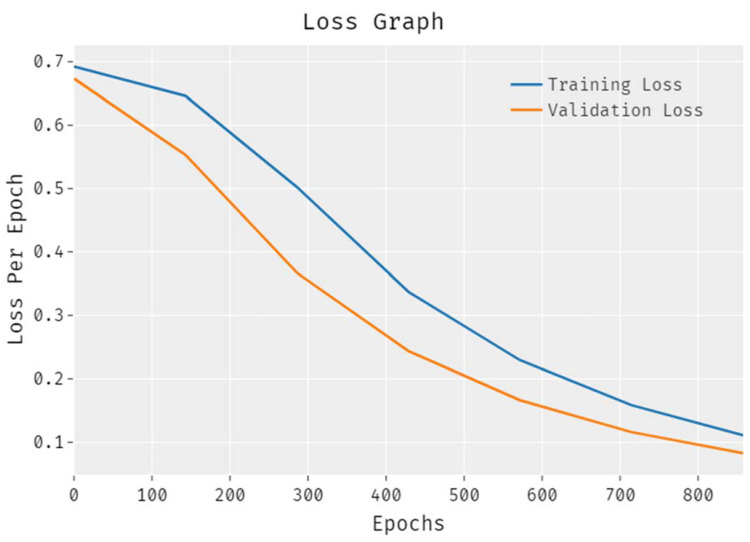
Loss graph analysis for the EDLCDS-BCDC technique.

The loss outcome analysis results accomplished by the proposed EDLCDS-BCDC technique on test data are portrayed in Figure 7. The results reveal that the EDLCDS-BCDC approach reduced the validation loss as compared to the training loss. It is also shown that the loss values were saturated with increasing numbers of epochs.

**Figure 8:**
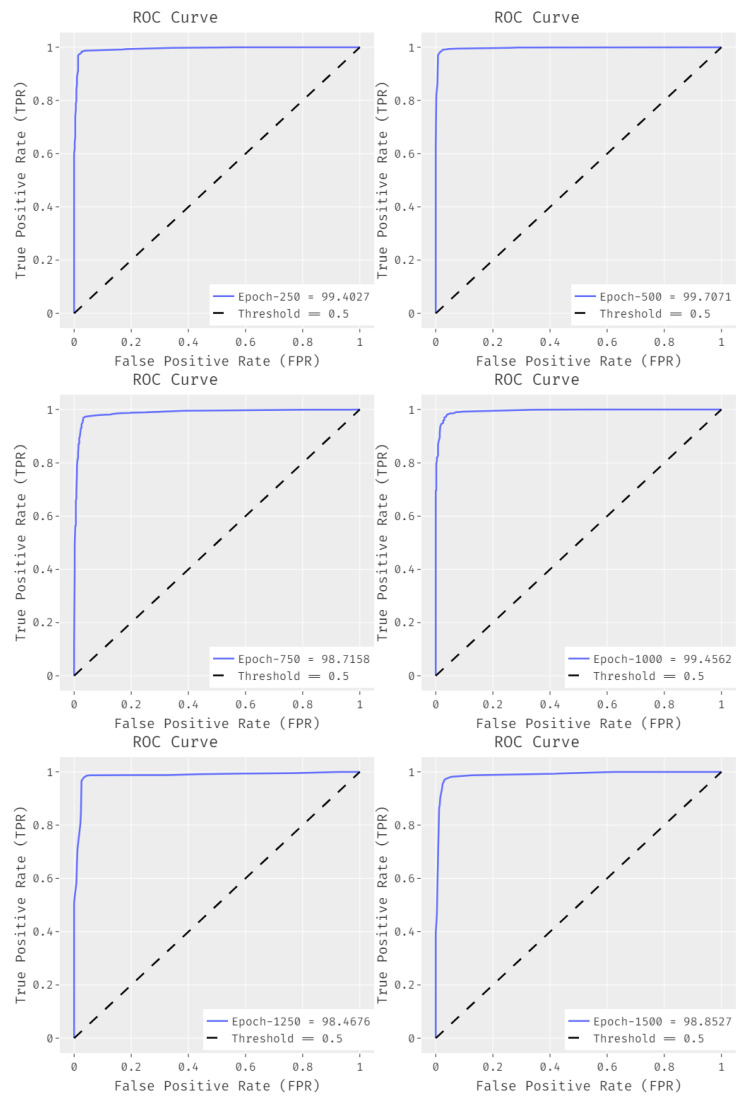
ROC analysis results for the EDLCDS-BCDC technique under distinct epochs.

Figure 8 illustrates the set of ROC curves obtained by EDLCDS-BCDC technique under distinct epochs. The results show that the proposed EDLCDS-BCDC technique achieved an increased ROC of 99.4027 under 250 epochs, 99.7071 under 500 epochs, 98.7158 under 750 epochs, 99.4562 under 1000 epochs, 98.4676 under 1250 epochs, and 98.8527 under 1500 epochs.

**Figure 9:**
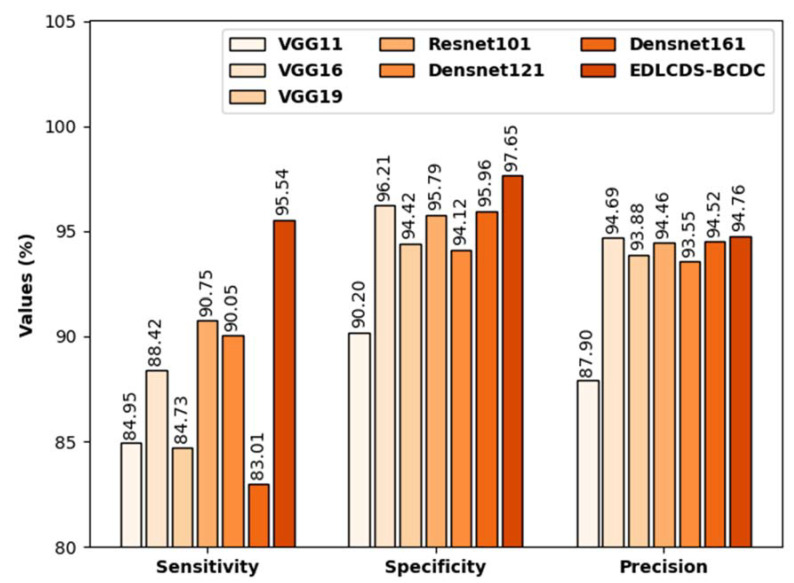
Comparative analysis of the EDLCDS-BCDC technique with recent methods.

Figure 9 contains the comparative analysis results, in terms of $sens_{y}$, $spec_{y}$, and $prec_{n}$, for the proposed EDLCDS-BCDC technique as well as other recent approaches [25]. The results indicate that the VGG19 and Densnet161 models obtained the lowest values of $sens_{y}$, $spec_{y}$, and $prec_{n}$.

In addition, the VGG11, Resnet101, and Densenet161 models produced slightly increased $sens_{y}$, $spec_{y}$, and $prec_{n}$ values. The VGG16 model accomplished reasonably good $sens_{y}$, $spec_{y}$, and $prec_{n}$ values of 84.42%, 96.21%, and 94.69%, respectively. However, the proposed EDLCDS-BCDC technique surpassed the available methods with the highest $sens_{y}$, $spec_{y}$, and $prec_{n}$ values of 84.95%, 90.20%, and 87.90%, respectively.

**Figure 10:**
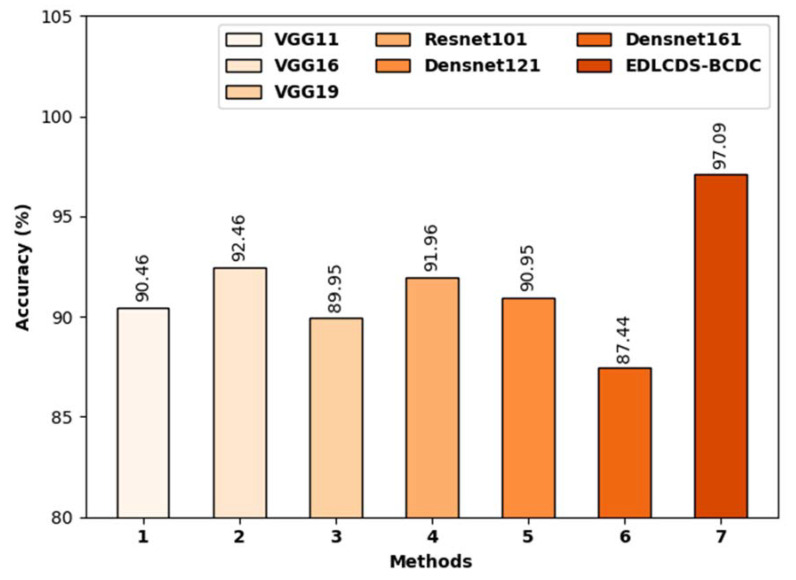
Accuracy analysis of the EDLCDS-BCDC technique compared with recent methods.

Figure 10 highlights the comparative analysis results, in terms of $accu_{y}$, accomplished by EDLCDS-BCDC and recent approaches [25]. The results indicate that both the VGG19 and Densnet161 models obtained low $accu_{y}$. In addition, the VGG11, Resnet101, and Densenet161 models produced slightly increased $accu_{y}$ values. Moreover, the VGG16 model accomplished a reasonable $accu_{y}$ of 92.46%. However, the proposed EDLCDS-BCDC technique surpassed all other available methods with the highest $accu_{y}$ of 97.09%.

The above-discussed results establish that the proposed EDLCDS-BCDC technique is a promising candidate for the recognition of breast lesions using USIs.

## 5. Conclusions

The current research work developed a novel EDLCDS-BCDC model to diagnose breast cancer using USIs. Primarily, USIs are pre-processed in two stages, namely noise elimination and contrast enhancement. These stages are followed by CKHA-KE based image segmentation, with ensemble DL-based feature extraction processes also being performed. Finally, the CSO-MLP technique is utilized to classify the images in terms of breast cancer either being present or not. Extensive experimental analyses were conducted using the proposed EDLCDS-BCDC technique on a benchmark database and the results were examined under distinct measures. The comparative results established the supremacy of the proposed EDLCDS-BCDC technique over existing methods. In future, deep instance segmentation techniques can be designed to enhance the detection rate of the EDLCDS-BCDC technique.

## Figures and Tables

**Table 1:** Analysis results of EDLCDS-BCDC technique with distinct epochs.

Classes	Sensitivity (%)	Specificity (%)	Precision (%)	Accuracy (%)
Epoch-250				
Benign	95.88	96.79	97.44	96.28
Malignant	95.24	97.54	93.46	96.92
Normal	95.49	98.61	93.38	98.08
Epoch-500				
Benign	96.57	95.63	96.57	96.15
Malignant	92.86	98.95	97.01	97.31
Normal	96.99	97.99	90.85	97.82
Epoch-750				
Benign	95.65	96.21	96.98	95.90
Malignant	91.90	98.25	95.07	96.54
Normal	98.50	97.68	89.73	97.82
Epoch-1000				
Benign	97.03	96.79	97.47	96.92
Malignant	94.76	98.77	96.60	97.69
Normal	96.24	98.30	92.09	97.95
Epoch-1250				
Benign	96.57	97.67	98.14	97.05
Malignant	94.29	97.72	93.84	96.79
Normal	96.24	98.30	92.09	97.95
Epoch-1500				
Benign	96.34	95.34	96.34	95.90
Malignant	92.86	98.25	95.12	96.79
Normal	96.24	98.45	92.75	98.08

**Table 2:** Average analysis results for the EDLCDS-BCDC technique under different measures.

No. of Epochs	Sensitivity (%)	Specificity (%)	Precision (%)	Accuracy (%)
Epoch-250	96.01	97.95	95.39	97.52
Epoch-500	95.47	97.52	94.81	97.09
Epoch-750	95.35	97.38	93.93	96.75
Epoch-1000	95.54	97.65	94.76	97.09
Epoch-1250	95.70	97.90	94.69	97.26
Epoch-1500	95.15	97.35	94.74	96.92

## Data Availability

Data sharing is not applicable to this article as no datasets were generated during the current study.
